# The cut-off values of dietary energy intake for determining metabolic syndrome in hemodialysis patients: A clinical cross-sectional study

**DOI:** 10.1371/journal.pone.0193742

**Published:** 2018-03-14

**Authors:** Tuyen Van Duong, Te-Chih Wong, Hsi-Hsien Chen, Tzen-Wen Chen, Tso-Hsiao Chen, Yung-Ho Hsu, Sheng-Jeng Peng, Ko-Lin Kuo, Chi-Sin Wang, I-Hsin Tseng, Yi-Wei Feng, Tai-Yue Chang, Chien-Tien Su, Shwu-Huey Yang

**Affiliations:** 1 School of Nutrition and Health Sciences, Taipei Medical University, Taipei, Taiwan; 2 Department of Nutrition and Health Sciences, Chinese Culture University, Taipei, Taiwan; 3 Department of Nephrology, Taipei Medical University Hospital, Taipei, Taiwan; 4 School of Medicine, Taipei Medical University, Taipei, Taiwan; 5 Department of Nephrology, Taipei Medical University- Wan Fang Hospital, Taipei, Taiwan; 6 Division of Nephrology, Department of Internal Medicine, Taipei Medical University- Shuang Ho Hospital, Taipei Medical University, Taipei, Taiwan; 7 Division of Nephrology, Cathay General Hospital, Taipei, Taiwan; 8 Division of Nephrology, Taipei Tzu-Chi General Hospital, Taipei, Taiwan; 9 School of Public Health, Taipei Medical University, Taipei, Taiwan; 10 Department of Family Medicine, Taipei Medical University Hospital, Taipei, Taiwan; 11 Nutrition Research Center, Taipei Medical University Hospital, Taipei, Taiwan; National Yang-Ming University, TAIWAN

## Abstract

Dietary energy intake strongly linked to dialysis outcomes. We aimed to explore the optimal cut-off point of energy intake (EI) for identification of metabolic syndrome (MetS) in hemodialysis patients. The cross-sectional data of 243 hemodialysis patients from multi-dialysis centers in Taiwan was used. The dietary intake was assessed by using the three-day dietary questionnaire, and a 24-hour dietary recall, clinical and biochemical data were also evaluated. The MetS was diagnosed by the Harmonized Metabolic Syndrome criteria. The receiver operating characteristic (ROC) curve was to depict the optimal cut-off value of EI for the diagnosis of MetS. The logistic regression was also used to explore the association between inadequate EI and MetS. The optimal cut-off points of EI for identifying the MetS were 26.7 kcal/kg/day for patients aged less than 60 years, or with non-diabetes, and 26.2 kcal/kg/day for patients aged 60 years and above, or with diabetes, respectively. The likelihood of the MetS increased with lower percentiles of energy intake in hemodialysis patients. In the multivariate analysis, the inadequate dietary energy intake strongly determined 3.24 folds of the MetS. The assessment of dietary EI can help healthcare providers detecting patients who are at risk of metabolic syndrome.

## Introduction

The end-stage renal disease (ESRD) has been steadily increased over the past decades and well-recognized as a heavy burden for every healthcare system in the world [1]. Taiwan experienced the highest number of hemodialysis patients in the world with 3093 dialysis per million population, 90% of patients receiving in-center hemodialysis [1]. However, the number of healthcare providers such as nephrologists, dietitians, or nutritionists has not increased to meet the greater demand of this group of patients in renal care [2].

Metabolic syndrome (MetS) showed the causal association with progressive decline in renal function [3]. MetS was reported with high prevalence in the end-stage renal disease (ESRD) patients undergoing hemodialysis, ranged from 61.0% diagnosed in Taiwan using criteria set by the adult treatment panel III (ATP-III) [4], to 75.3% diagnosed in Brazil according to the Harmonizing the Metabolic Syndrome (HMetS) criteria [5]. MetS has been practically established as the risk factor for cardiovascular disease, type 2 diabetes, and increase in all-cause [5–10], and predicted the risk of hospitalization [11]. On the other hand, the progression of renal disease may lead to elevated blood pressure, hypertriglyceridemia, and other metabolic alterations which may further add to the incidence and prevalence of metabolic syndrome [12]. The progressive decline of renal function even induce the onset of insulin resistance and diabetes independent of previous diabetic and nutritional status [10]. In addition, specific treatment modalities may have a negative metabolic effect favoring the onset of metabolic abnormalities [12].

The detection of MetS and nutritional interventions were the most critical recommendations, in order to have adequate interventions to reduce the unfavorable consequences [13,14]. Dietary approaches have been recognized as the effective therapy to prevent several risk factors and its unfavorable consequences in patients with chronic diseases, especially to prevent metabolic complications, and reduce the metabolic syndrome alteration [15–18]. A potential justification for the increased energy dietary intake was recommended in the National Kidney Foundation-Kidney Disease Outcomes Quality Initiative (K/DOQI) guidelines [19,20].

The inadequate energy intake (IEI) was high prevalence, accounted for about two third of hemodialysis patients [21,22]. The randomized controlled trial on patients with metabolic syndrome concluded that dietary approaches reduced most of the metabolic risk factors [14]. Dietary energy intake has been well recognized as a determinant of metabolic syndrome. However, there has been none of the studies estimate the cut-off value of energy intake, which can detect the MetS.

In order to face and overcome these critical problems of limited human resource and to lower the cost of diagnostic tests and treatment, dietary intake assessment has become vitally important to identify metabolic syndrome in hemodialysis patients. The current study used the data from multi-dialysis centers in Taiwan to explore the optimal cut-off point of energy intake for identification of MetS in hemodialysis patients.

## Methods

### Study design and patient population

The hemodialysis (HD) patients were recruited from a clinical cross-sectional study, which was conducted between September 2013 and November 2016. The data of 243 HD patients in hemodialysis centers from five hospitals in Taiwan including 58 from Taipei Medical University Hospital, 55 from Taipei Tzu-Chi Hospital, 52 from Taipei Medical University–Wan Fang Hospital, 42 from Cathay General Hospital, 36 from Taipei Medical University–Shuang Ho Hospital. The sample size was adequate for a clinical observational design.

Patients who aged above 20 years, received thrice-weekly hemodialysis treatment for at least 3 months, adequate dialysis quality (equilibrated Kt/V ≥ 1.2 g/kg/day) were recruited. Patients who had one of the following criteria were excluded: who diagnosed with edema, pregnancy, amputation, hyperthyroidism, hypothyroidism, malignancy, received tube feeding, exhibited hepatic failure or cancer, hospitalized within one month prior to the recruitment, or were scheduled for surgery. In the current study, patients have not been in diet control for overweight or obesity. They have been advised to follow the K/DOQI guidelines [23], and healthy eating guidelines in Taiwan [24,25].

### Dietary energy intake

The dietary intake was evaluated via the three-day dietary record (one day of hemodialysis, one day of non-hemodialysis, and one day in the weekend), and a 24-hour dietary recall with common household measuring utensils was also administered as the means to confirm the data, which described in details elsewhere [26,27]. In brief, patients were asked about meal time, meal location, food names, brand names, ingredients, portion or weight of foods, and the different cooking methods and oils used. The nutrients were then analyzed e-Kitchen software (Nutritionist Edition, Enhancement plus 3, version 2009, Taichung, Taiwan).

The guidelines of National Kidney Foundation-Kidney Disease Outcomes Quality Initiative (NKF-K/DOQI) recommended that the optimal targets for dietary energy intake in hemodialysis patient were ≥ 35 kcal/kg/day if age < 60, and ≥ 30 kcal/kg/day if age ≥ 60, respectively [19,20]. Patients consumed less than those cut-off points were classified as inadequate dietary energy intake.

### Clinical and laboratory data

The body compositions including height (cm), weight (kg), body mass index, BMI (kg/m^2^), and waist circumference, WC (cm) were measured by bioelectrical impedance analysis (BIA) (InBody S10, Biospace, Seoul, Korea), the measurement procedures was performed according to manufacturer guidelines as described details elsewhere [28]. The hemodialysis vintage, comorbidities (diabetes mellitus, hypertension, cardiovascular diseases, and others), systolic blood pressure, diastolic blood pressure before each hemodialysis session were also assessed by reviewing patients’ medical records. The comorbidity index was calculated by adapted the Charlson comorbidity index for end-stage renal disease patients [29].

Physical activity was assessed by the short version of the International Physical Activity Questionnaire (IPAQ-SF) with 7 items was used in this study. The total time spent on physical activity was the sum of total minutes over last seven days spent on the vigorous activity, moderate activity, walking, and sitting multiplied by 8.0, 4.0, and 3.3, 1.0, respectively [30]. The time spent on physical activity was then transformed into the metabolic equivalent task minute per week (MET- min/wk), to create MET scores, this scoring method was used in several studies [31].

The serum level of high-sensitive C-reactive protein (hs-CRP), fasting plasma glucose (FPG), triglyceride (TG), and high-density lipid cholesterol (HDL-C) were archived from laboratory tests. The elevated level of high-sensitive C-reactive protein (hs-CRP) was classified as hs-CRP > 0.5 mg/dl [32]. The high sensitive C-reactive protein was seen as the most sensitive biomarker of the systemic inflammation which strongly associated with MetS than other biomarkers [33,34]. The hs-CRP was then assessed in the current study as the inflammation marker.

### Diagnosis of metabolic syndrome

Metabolic syndrome (MetS) was defined by Harmonizing Metabolic Syndrome definition (HMetS) which patients had three or more metabolic abnormalities: elevated waist circumference (WC ≥ 90 cm for men, ≥ 80 cm for women), high serum triglyceride (TG ≥150 mg/dL), low HDL cholesterol (HDL-C <40 mg/dL in men or <50 mg/dL in women), high blood pressure (blood pressure ≥ 130 mmHg systolic or ≥ 85 mmHg diastolic), impaired fasting glucose (elevated fasting plasma glucose, FPG ≥ 100 mg/dL[35], or diagnosed with type 2 diabetes mellitus) [36].

### Statistical analysis

The receiver operating characteristic (ROC) curve was used to depict the optimal cut-off value of energy intake for the diagnosis of HMetS. The area under the curve (AUC), and 95% confident interval, sensitivity, and specificity were reported for the overall sample, male, female, aged less than 60 years, 60 years and above, patients with diabetes mellitus (DM), and without DM. The ROC curves were interpreted as the probability that the EI values can correctly discriminate patients with HMetS from those EI values without HMetS, where 0.5 is chance discrimination and 1.0 is perfect discrimination [37].

To determine the optimal cut-off value, two common methods were used, which was the point on the ROC curve closest to (0,1) and the maximum Youden index (*J*) [38]. The Youden index (*J*) was calculated as [sensitivity- (1-specificity)], and the point with shortest distance value from the point (0,1) was calculated as [(1—sensitivity)^2^ + (1—specificity)^2^] [37,38]. In addition, the positive likelihood ratio (PLR), which summarizes how likely patients with the HMetS were to have a specified value of EI compared with patients without the HMetS. The PLR values were calculated as sensitivity/(1-specificity) [37]. The analyses were performed for the overall sample, and subgroups of male, female, age less than 60 years, 60 years and above, patients with DM, and without DM.

The correlation of energy intake, carbohydrate, protein, and total fat intake with MetS and its components were analyzed by Spearman test. Finally, the logistic regression models were used to examine the association between energy intake and metabolic syndrome in hemodialysis patients, by using newly developed cut-off point and targeted dietary energy intake recommended by NKF-K/DOQI. Since body mass index, hemodialysis vintage, physical activity level, and high sensitive C-reactive protein were reported in number of studies that they associated with metabolic syndrome in hemodialysis patients [33,39–41], which can confound the association between energy intake and MetS. Therefore, these factors will be adjusted in the multivariate analysis.

All statistical analyses were performed with the SPSS for Windows version 20.0 (IBM Corp., New York, USA). The significant level was set at P < 0.05.

### Ethical consideration

The study was ethically approved by Taipei Medical University Joint Institutional Review Board (TMU-JIRB No. 201302024), Cathay General Hospital (CGH-OP104001), and Taipei Tzu-Chi Hospital (04-M11-090). The study has been conducted according to the principles expressed in the Declaration of Helsinki. All patients involved in the study have signed the informed consent statement which the subject confidentiality is upheld (S1 File).

## Results

### Patients’ characteristics

The mean and standard deviation of age was 61.4 ± 11.2 years, 54.3% men, 40.3% overweight and obese, daily dietary energy intake, percentage of carbohydrate, protein, and total fat intake were 28.0 ± 9.4 (kcal/kg), 48.7 ± 9.3, 15.1 ± 3.5, and 35.9 ± 8.6, respectively. The median (IQR) of age among patients less than 60 years old, and 60 years old and above were 53.0 (49.0, 56.0), and 68 (63.0, 74.5), respectively. The prevalence of diagnosed type 2 diabetes mellitus, impaired fasting glucose, elevated waist circumference, high triglyceride, low HDL-Cholesterol, and high blood pressure were 39.5%, 66.3%, 33.7%, 37.2%, 62.2%, and 81.1%, respectively. Among patients, 55.6% were diagnosed with metabolic syndrome (Table 1).

**Table 1:** Characteristics of hemodialysis patients.

Variables	Total sample (N = 243)	
	Mean ± SD	n (%)
Age, years	61.4 ± 11.2	
Age less than 60 years, median (IQR)	53.0 (49.0, 56.0)	
Age 60 years and above, median (IQR)	68.0 (63.0, 74.5)	
Male gender,		132 (54.3)
Diagnosed T2DM		96 (39.5)
Hemodialysis vintage, median (IQR) years	4.2 (2.2–7.9)	
Charlson Comorbidity Index, median (IQR)	2.0 (1.0–3.0)	
Physical activity, MET-min/wk	4557 ± 1945	
Body mass index (kg/m^2^)	23.6 ± 3.8	
Overweight/Obese (BMI ≥ 24.0 kg/m^2^)		98 (40.3)
hs-CRP, median (IQR) mg/dL	0.2 (0.1–0.5)	
Elevated level (hs-CRP > 0.5 mg/dL)		65 (26.7)
Dietary energy intake, kcal/kg/day	28.0 ± 9.4	
Carbohydrates (%EI)	48.7 ± 9.3	
Protein (%EI)	15.1 ± 3.5	
Total fat (%EI)	35.9 ± 8.6	
Metabolic parameters		
Fasting plasma glucose (mg/dL)	123.9 ± 48.9	
IFG (FPG ≥ 100 mg/dL, or previously diagnosed T2DM)		161 (66.3)
Waist circumference (cm)	82.4 ± 10.4	
Elevated WC (≥ 90 cm for men, ≥ 80 cm for women)		82 (33.7)
Triglyceride (mg/dL)	147.1 ± 99.2	
High TG (TG ≥ 150 mg/dL)		90 (37.2)
HDL-C (mg/dL)	39.8 ± 23.8	
Low HDL-C (<40 mg/dL for men, < 50 mg/dL for women)		138 (62.2)
Systolic BP (mmHg)	149.6 ± 24.4	
Diastolic BP (mmHg)	76.2 ± 12.7	
High BP (BP ≥ 130/85 mmHg)		197 (81.1)
HMetS		135 (55.6)

Abbreviations: SD, Standard deviation; IQR, Interquartile range from quartile 1 to quartile 3; MET, metabolic equivalent minute/ week; hs-CRP, high sensitive C-reactive protein; EI, energy intake; IFG, Impaired fasting glucose; FPG, fasting plasma glucose; T2DM, type 2 diabetes mellitus; WC, waist circumference; TG, triglyceride; HDL-C, high-density lipoprotein cholesterol; BP, blood pressure; HMetS, harmonized metabolic syndrome.

### Receiver operating characteristic curve analysis

**Fig 1:**
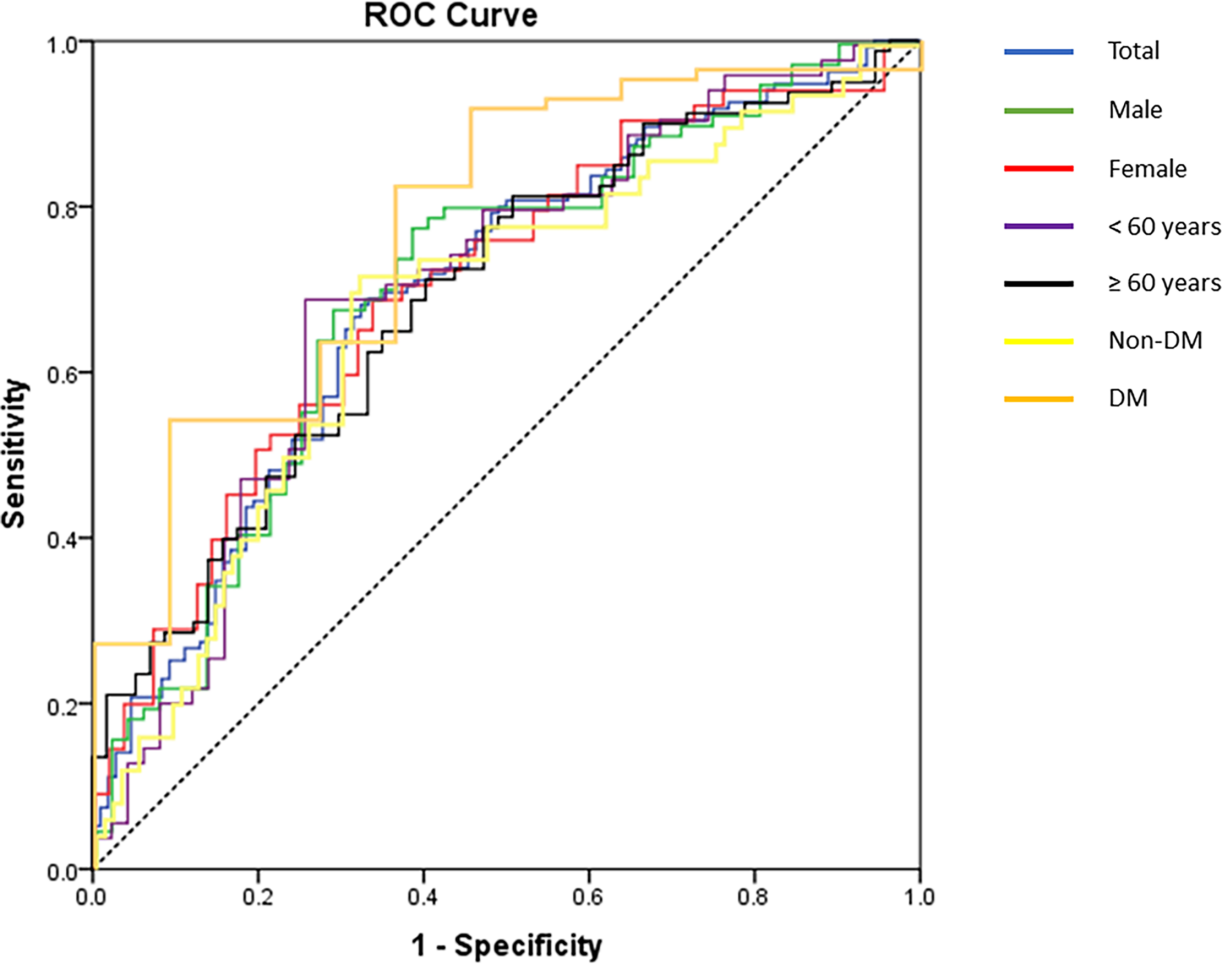
The receiver operating characteristic (ROC) curve of energy intake predicting the harmonized metabolic syndrome in hemodialysis patients. Abbreviations: DM, diabetes mellitus.

**Fig 2:**
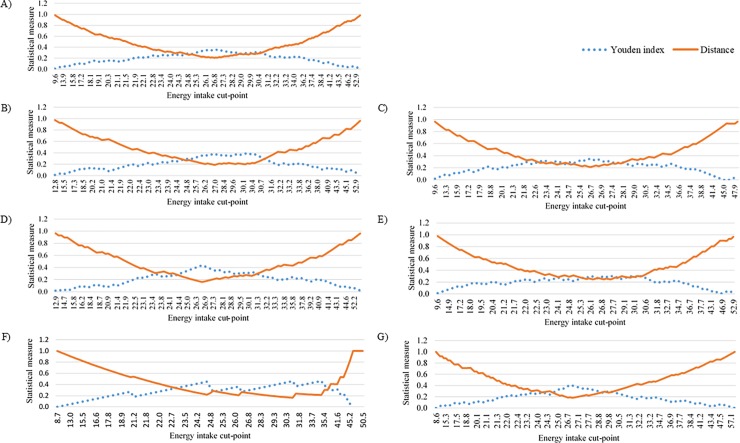
The optimal cut point of energy intake for predicting the hamonized metabolic syndrome. The panel (A) shows results in total sample, (B) in male, (C) in female, (D) in aged < 60 years, (E) in aged 60 years and above, (F) in non-diabetes mellitus, (G) in diabetes mellitus.

Results from ROC curve analysis showed that energy intake lower or equal to 26.7 kcal/kg was as a determinant of metabolic syndrome in the overall sample, in male, female, patients aged less than 60 years, and without diabetes. The cut-off point was slightly lower to 26.2 kcal/kg for patients aged 60 years and above, and with diabetes. In the overall sample, results showed 67% sensitivity, 69% specificity, AUC 0.70 (95%CI, 0.63–0.76, P < 0.001), with a positive likelihood ratio of 2.10, highest Youden index of 0.35, and shortest distance to the point (0,1) of 0.21. The final cut-off values selected by Youden index and shortest distance to the point (0,1) were the same (Table 2, Figs 1 and 2).

**Table 2:** The area under the ROC curve, sensitivity, specificity, positive likelihood ratio, Youden index, distance to the point (0,1), and cut-off values of energy intake to predict HMetS.

	Total n = 243	Male n = 132	Female n = 111	< 60 years n = 106	≥ 60 years n = 137	Non-DM n = 147	DM n = 96
Area under the ROC curve, AUC (95%CI)	0.70 (0.63–0.76)	0.70 (0.61–0.80)	0.71 (0.61–0.80)	0.70 (0.60–0.80)	0.69 (0.60–0.78)	0.68 (0.59–0.77)	0.77 (0.63–0.92)
Sensitivity	0.67	0.66	0.69	0.69	0.65	0.70	0.64
Specificity	0.69	0.71	0.66	0.74	0.65	0.69	0.73
Positive Likelihood Ratio	2.10	2.30	2.04	2.71	1.85	2.26	2.33
Highest Youden index	0.35	0.37	0.35	0.44	0.30	0.39	0.36
Shortest distance to the point (0,1)	0.21	0.20	0.21	0.16	0.25	0.19	0.21
Cut-point C_*J*_	26.7	26.7	26.7	26.7	26.2	26.7	26.2
Cut-point C*	26.7	26.7	26.7	26.7	26.2	26.7	26.2

Abbreviations: HMetS, harmonized metabolic syndrome; DM, diabetes mellitus; AUC, area under the ROC curve; CI, confident interval; C_*J*_, the optimal cut-off point identified by maximum Youden index value; C*, the optimal cut-off point identified by the point closest to the (0,1) point.

**Fig 3:**
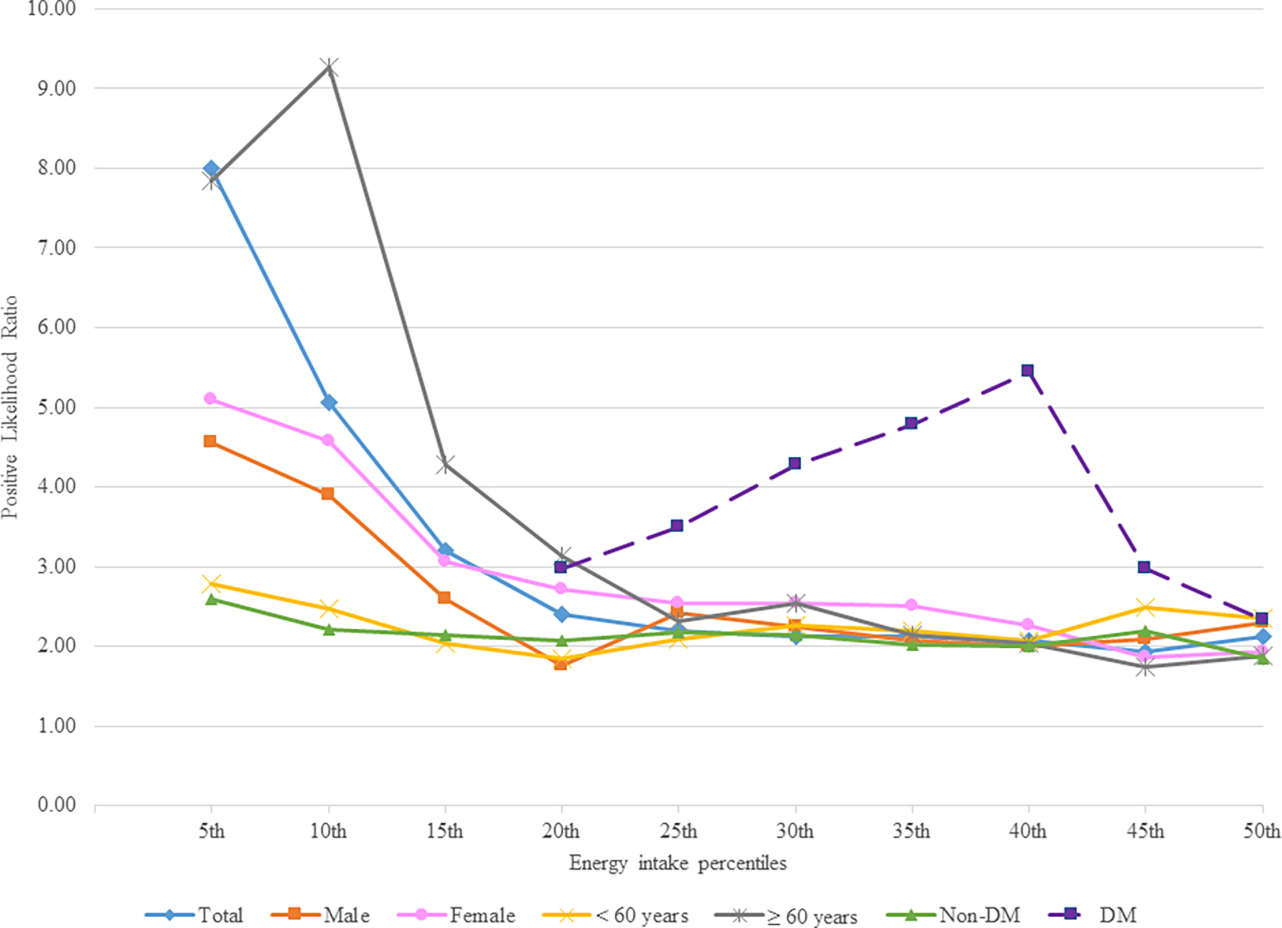
Positive likelihood ratios of different energy intake percentiles for prediction of harmonized metabolic syndrome. Abbreviations: DM, diabetes mellitus.

The likelihood of HMetS mostly increased with the decreased percentiles of energy intake from 50^th^ to 5^th^ percentiles. The likelihood ratios of HMetS of overall sample, and subgroups were slightly increased from 50^th^ to 20^th^ percentiles, and dramatically increased from 20^th^ to 5^th^ percentiles. The highest likelihood ratios of 8.00, 4.55, 5.09, 2.78, 2.59, 9.26, and 5.44 for total sample, male, female, age less than 60 years, and without diabetes at 5^th^ percentile, age 60 and above at 10^th^ percentile, and with diabetes at 40^th^ percentile, respectively (Fig 3).

### Association between inadequate energy intake and metabolic syndrome

Results of a spearman correlation analysis show that higher energy intake was significantly associated with lower prevalence of metabolic syndrome and its components (impaired fasting glucose, elevated waist circumference, high triglyceride, low HDL-Cholesterol). Carbohydrate, protein, and total fat intake did not significantly illustrate the association with metabolic syndrome and the metabolic components (Table 3).

**Table 3:** The correlation of energy intake, carbohydrate, protein, and total fat with metabolic syndrome and metabolic components.

	Energy intake	Carbohydrate (%EI)	Protein (%EI)	Total Fat (%EI)	HMetS	IFG	Elevated WC	High TG	Low HDL-C	High BP
Energy intake	1.00									
Carbohydrate	-.16*	1.00								
Protein	-.13*	-.37**	1.00							
Total Fat	.20**	-.90**	.08	1.00						
HMetS	-.34**	-.02	.01	.01	1.00					
IFG	-.28**	.03	-.03	-.06	.55**	1.00				
Elevated WC	-.37**	-.09	.05	.07	.39**	.14*	1.00			
High TG	-.24**	.03	-.10	-.001	.55**	.22**	.28**	1.00		
Low HDL	-.16*	.04	.01	-.08	.57**	.12	.18**	.36**	1.00	
High BP	-.01	-.04	.08	.02	.22**	.17**	-.12	.001	-.11	1.00

* Correlation is significant at the 0.05 level (2-tailed).

** Correlation is significant at the 0.01 level (2-tailed).

Abbreviations: EI, energy intake; HMetS, harmonized metabolic syndrome; IFG, Impaired fasting glucose; WC, waist circumference; TG, triglyceride; HDL-C, high-density lipoprotein cholesterol; BP, blood pressure.

The inadequate energy intake was then classified as energy intake lower than the cut-off point of 26.7 kcal/kg for patients aged less than 60 years, and 26.2 kcal/kg for patients aged 60 years and above, named inadequate energy intake for determining the metabolic syndrome (or IEI-M). The prevalence of inadequate energy intake (IEI-M) was 50.6%, and prevalence of IEI defined by National Kidney Foundation Kidney Disease Outcomes Quality Initiative (IEI-K/DOQI) was 71.2% (Table 4).

**Table 4:** Inadequate energy intake determines the metabolic syndrome in hemodialysis patients.

Energy intake	Total sample	Model 1			Model 2		
	n(%)	B ± SE	OR (95%CI)	P value	B ± SE	OR (95%CI)	P value
IEI-M ^a^	123 (50.6)	1.51 ± 0.28	4.55 (2.64, 7.83)	< 0.001	1.18 ± 0.32	3.24 (1.74, 6.05)	< 0.001
IEI-K/DOQI ^b^	173 (71.2)	1.32 ± 0.30	3.75 (2.08, 6.76)	< 0.001	0.92 ± 0.34	2.50 (1.28, 4.87)	< 0.01

Model 1 included dietary intake and metabolic syndrome.

Model 2 adjusted for age, gender, body mass index, hemodialysis vintage, physical activity level, and high sensitive C-reactive protein.

^a^ Inadequate energy intake diagnosed by new cut-off point for predicting metabolic syndrome in hemodialysis patients.

^b^ Inadequate energy intake diagnosed by the National Kidney Foundation Kidney Disease Outcomes Quality Initiative (K/DOQI) Workgroup.

Abbreviations: β, the standardized coefficient; SE, standard error; OR, Odd ratio; CI, confident interval.

In the bivariate analysis, IEI was significantly associated with higher prevalence of metabolic syndrome with OR = 4.55, 95% CI, 2.64–7.83, P < 0.001, and OR = 3.75, 95% CI, 2.08–6.76, P < 0.001, for IEI-M, and IEI- K/DOQI, respectively. After controlling for age, gender, body mass index, hemodialysis vintage, physical activity level, and high sensitive C-reactive protein, the association remained significant with OR = 3.24, 95% CI, 1.74–6.05, P < 0.001, and OR = 2.50, 95% CI, 1.28–4.87, P < 0.01, for IEI-M, and IEI- K/DOQI, respectively (Table 4).

## Discussion

The results of current study demonstrated the optimal cut-off points of energy intake for determining the MetS were 26.7 kcal/kg/day, and 26.2 kcal/kg/day, which were lower than the K/DOQI recommendation level for energy intake in hemodialysis patients of 35 kcal/kg/day, and 30 kcal/kg/day, for patients aged less than 60 years, and 60 years and above, respectively [20]. The wide distribution range of age between two age groups that could partly explain the wide range of difference between the highest likelihood ratios (2.78 versus 9.26) for age less than 60 years at 5th percentile, and age 60 and above at 10th percentile, respectively. The cut-off values of energy intake among patients without and with diabetes mellitus were 26.7 kcal/kg/day, and 26.2 kcal/kg/day, respectively. The positive likelihood of having MetS among DM patients were extreme high, and cannot be calculated from 5^th^ percentile to 20^th^ percentile of energy intake as the values of “1-specificity” are closed to zero. Among DM patients, the positive likelihood ratio (PLR) was increased from 2.98 at 20^th^ percentile to 5.44 at 40^th^ percentile, and decreased to 2.33 at 50^th^ percentile. In overall, the PLR of having MetS among DM patient was decreased by the increased percentile of energy intake among hemodialysis patients.

To harmonize with K/DOQI guideline for clinical practice, the energy intake can be classified into three levels, as severely inadequate energy intake with EI < 26.7, and < 26.2, moderate inadequate energy intake with 26.7 ≤ EI < 35, and 26.2 ≤ EI < 30, and adequate energy intake with EI ≥ 35, and ≥ 30, for patients aged less than 60 years, and 60 years and above, respectively.

The results demonstrated that energy intake was well established indicator among dietary components which associated with MetS and its components. In addition, the likelihood of the MetS increased with lower percentiles of energy intake in hemodialysis patients. In multivariate regression analysis, the inadequate dietary energy intake related to a higher odd of the MetS, and strongly determined 3.24, and 2.50 folds of harmonized metabolic syndrome (HMetS) via newly developed cut-off points, and those recommended by K/DOQI guideline, respectively. This could be explained that the energy balance can be disrupted in patients with inadequate energy intake, which related to a number of disorders such as risks of cardiovascular diseases, metabolic syndrome [42]. On the other hand, patients who had the adequate consumption of energy-enriched meal while receiving hemodialysis can strongly improve the whole body protein balance and in turn, improve the dialysis outcomes [43].

In addition, the prevalence of inadequate energy intake was high, about two third of HD patients in the current study, which was in the line with previous studies [21,22]. On the other hand, the metabolic syndrome was common in HD patients in present study (55.6% HMetS), which were lower than that in Brazil (74.5%) using diagnostic criteria from Harmonizing Metabolic Syndrome [41], and in the United States (69.3%) using NCEP-ATP III [44]. In HD patients, adequate energy dietary intake was recommended in the K/DOQI guidelines [19,20], to reduce the risk of metabolic syndrome, and improve the hemodialysis outcomes.

The current study presented with a number of limitations. Firstly, dietary intake was subjectively assessed. Fortunately, patients were interviewed for three different days and confirmed by 24-hour recall dietary questionnaire. Secondly, with the cross-sectional nature, the inferences of causal relationship should be cautious, regarding dietary energy intake and the development of metabolic abnormalities and the metabolic syndrome. The study has the strengths including the use of precise and direct measurement of body composition by BIA, and biochemical parameters were examined by standardized laboratory tests. Future studies with different designs were suggested to measure the longitudinal data of energy intake and to investigate the association between dietary intake and metabolic syndrome.

## Conclusions

The study demonstrated that the optimal cut-off points of energy intake for determining the MetS among patients aged less than 60 years, or without DM were 26.7 kcal/kg/day, and among patients aged 60 years and above, or with DM were 26.2 kcal/kg/day, respectively. The likelihood of the MetS increased with lower percentiles of energy intake in hemodialysis patients. The inadequate energy intake significantly associated with the higher odd of the MetS. The results indicated that hemodialysis patients with inadequate energy intake should be closely followed up, in order to identify the risks of the metabolic syndrome and have adequate examinations and treatments.

## Supporting information

S1 File Ethical approvals. (DOCX)
