# Supplementary material for: The cut-off values of dietary energy intake for determining metabolic syndrome in hemodialysis patients: A clinical cross-sectional study
Source: PLoS One. 2018 Mar 14;13(3):e0193742. doi: 10.1371/journal.pone.0193742 (PMC5851580; doi:10.1371/journal.pone.0193742)

**S1 file. Ethical Approvals**

Taipei Medical University Joint Institutional Review Board


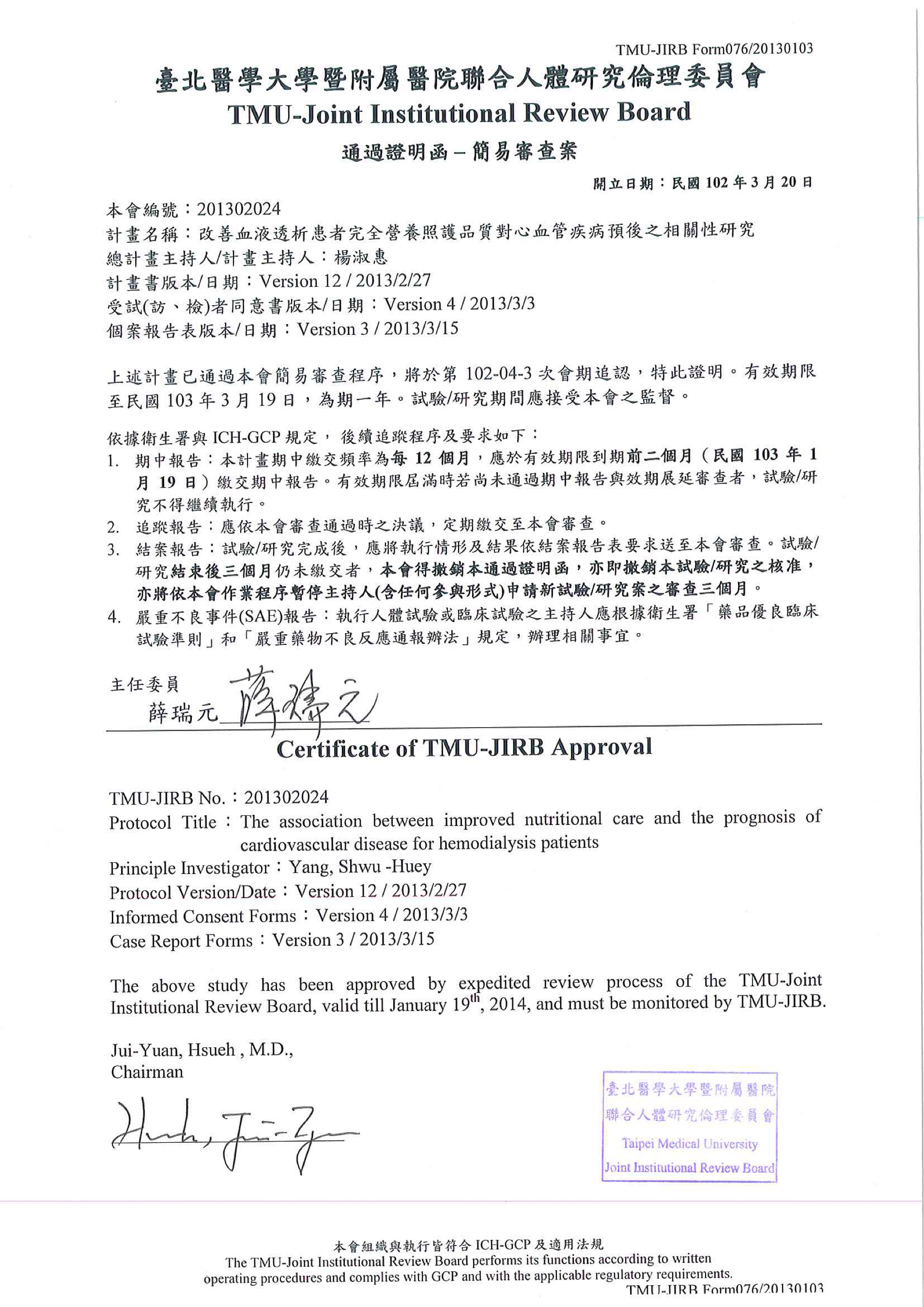


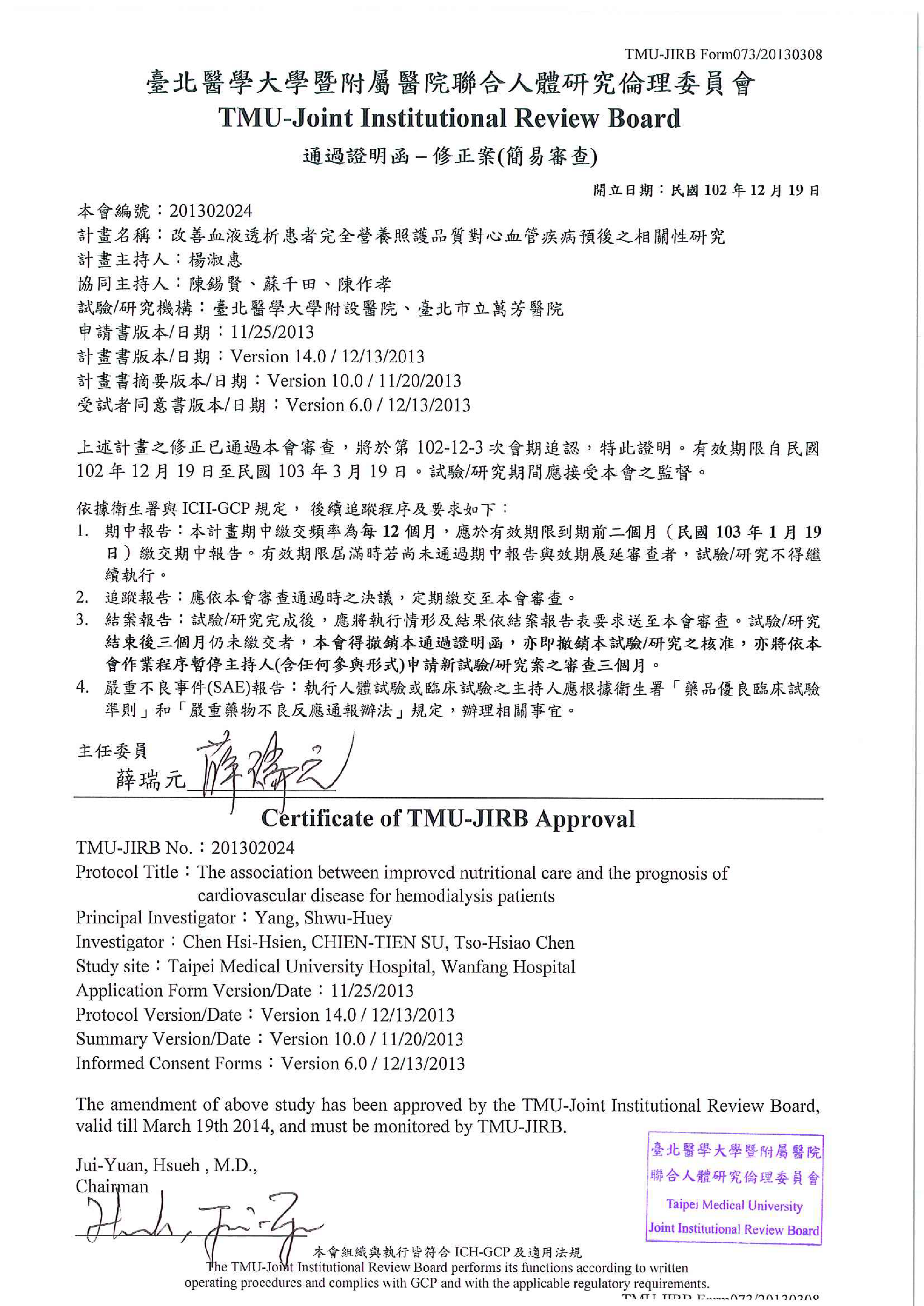


Institutional Review Board of the Cathay General Hospital


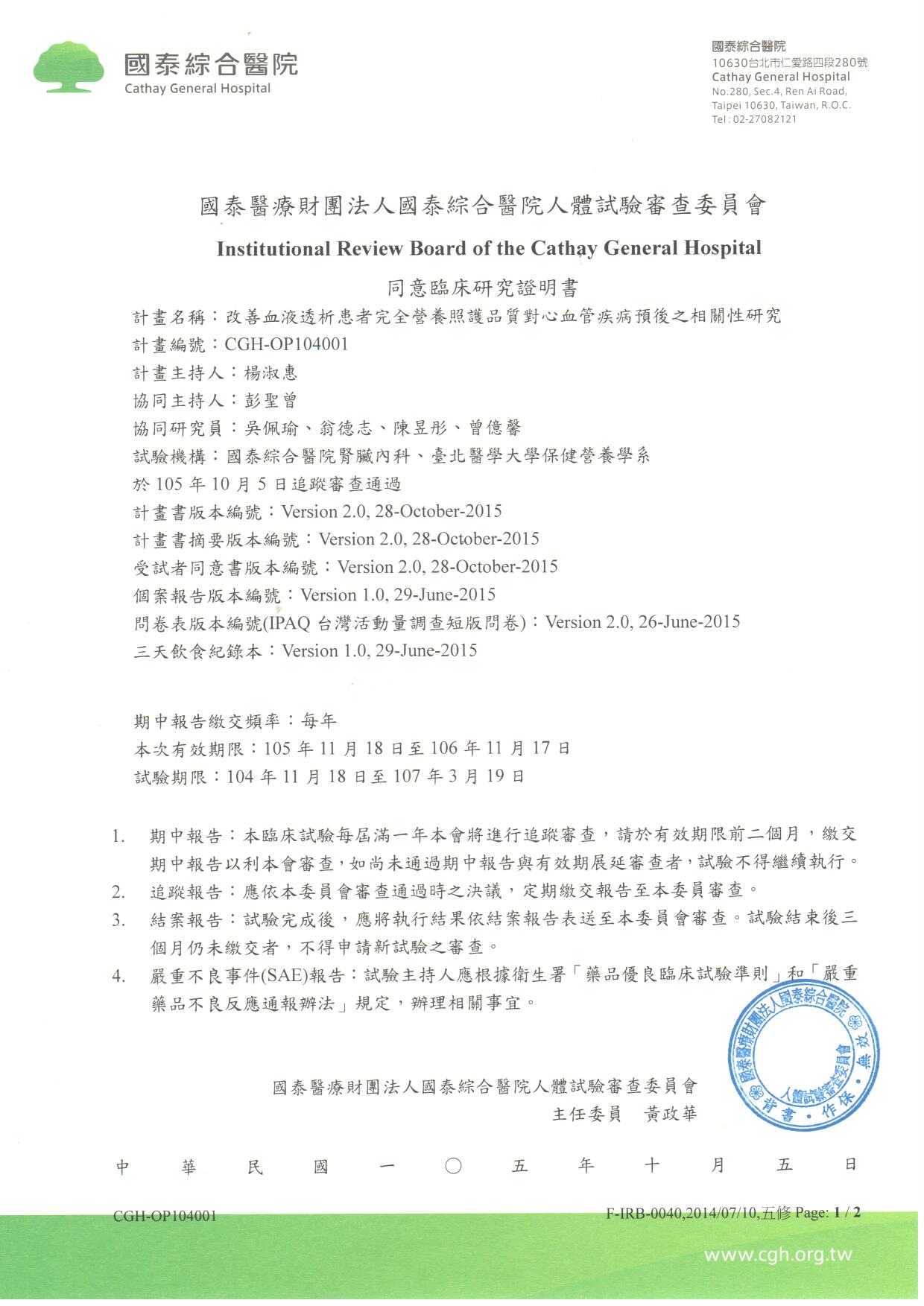


Institutional Review Board of the Taipei Tzu-Chi Hospital


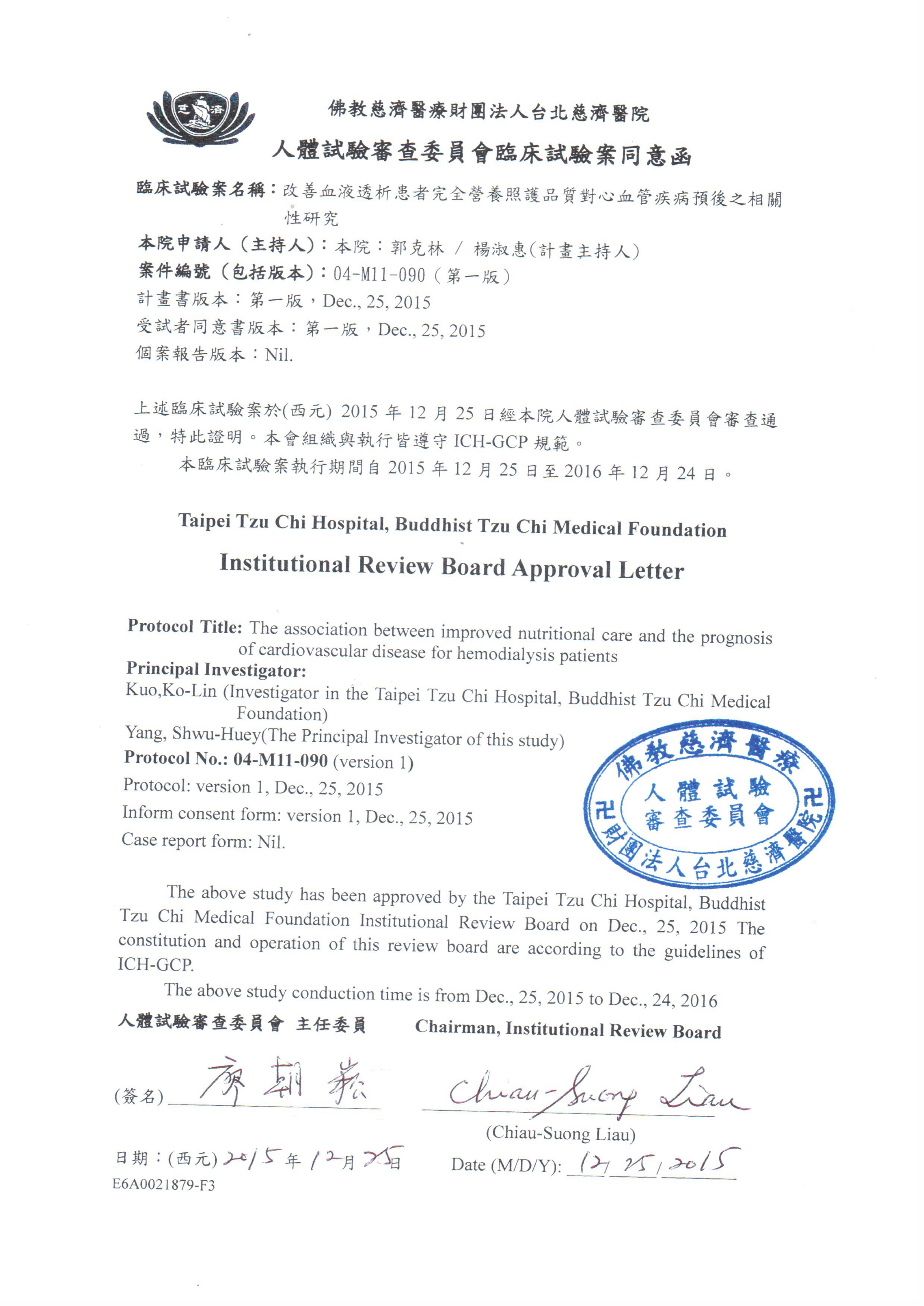

Supplement: S1 File — (DOCX) [file pone.0193742.s001.docx]
